# Examination of Chronic Sorrow Among Parents of Children With Disabilities: Cross-Sectional Study

**DOI:** 10.2196/65754

**Published:** 2025-07-02

**Authors:** Samaa Al Anazi, Naseem Alhujaili, Dina Sinqali, Ftoon Al Heej, Lojain Al Somali, Samaher Khayat, Talah Ramboo

**Affiliations:** 1 King Abdullah International Medical Research Center, Jeddah, Saudi Arabia; 2 College of Nursing - Jeddah, KSAU, KAMC, National Guard Health Affairs, PO Box 9515, Jeddah, 21423, Saudi Arabia, +966 12 224-46192 ext 46154, +966 12 224-46224 ext 46154; 3 Department of Medicine, Division of Psychiatry, Faculty of Medicine in Rabigh, King Abdulaziz University, Jeddah, Saudi Arabia

**Keywords:** chronic sorrow, sadness, parent, disability, pediatric, infant, neonatal, children, youth, adolescence, Saudi Arabia

## Abstract

**Background:**

Parents of children with disabilities face many challenges when providing care, along with persistent worry and fear about the child’s health outcomes and the impact of the disability on their lives. These parents experience stressful situations and face many emotions, one of which is chronic sorrow (CS). Therefore, the theory of CS was introduced to examine and measure feelings of CS among parents. Little research has been conducted with Saudi parents with a child with disabilities and the utilization of CS theory in this population is limited.

**Objective:**

This study aims to examine the application of CS theory on parents of children with disabilities in Saudi Arabia.

**Methods:**

A cross-sectional design was used to obtain data from 89 participants who are parents of children with disabilities. A web-based questionnaire was distributed to measure CS.

**Results:**

The study examined the concepts within CS theory. The concept of loss experience yielded a moderately high score (mean 3.3, SD 1.10); of all the variables measuring loss experience, parents scored high in feeling sad when thinking about their child’s disability (mean 3.9, SD 1.24). Parents also reported a moderately high score (mean 3.3, SD 1.06) for the concept of disparity, specifically when their child does not meet the same developmental milestones as their peers (mean 3.8, SD 1.30). Feelings of CS also displayed a moderately high score (mean 3, SD 0.87), with the periodic nature of CS scoring the highest within the concept of CS (mean 3.6, SD 1.16). In addition, internal and external management methods that parents believe are effective were examined. Internal management of CS was believed to be of high importance (mean 4.6, SD 0.33), specifically the belief of fatalism (mean 4.8, SD 0.50). Parents also viewed external management as important in navigating their emotions (mean 4.5, SD 0.42), specifically social support from family and the community (mean 4.7, SD 0.55). This study identified strong positive relationships between sorrow and loss experience and disparity (both *r*=0.765 and *P*<.001). Lastly, the study found no relationship between CS and time elapsed since parents received their child’s diagnosis (*r*=−0.009; *P*=.94).

**Conclusions:**

This study applied the theory of CS to the parents of children with disabilities and they reported feelings of loss, disparity, and CS. Therefore, this population should be screened and provided with parental emotional care. Interventions to enhance parental mental health and well-being and support CS management should be developed and used by health care workers. Parental acceptance of their child’s disability does not mean the absence of CS, as it is part of the normal grieving process. Anticipating CS triggers and applying internal and external management are essential to improving parental mental health and child health outcomes.

## Introduction

### Background

In 2017, the Saudi General Authority of Statistics issued a detailed report of people with disabilities who reside in Saudi Arabia, noting that a total of 1,445,723 people with varying degrees of disability (mild, moderate, or severe) reside in the country, of which 52.2% were males and 47.7% were females [1]. This illustrates that people with disabilities represent a substantial portion of the Saudi community and more attention should be directed to them and their families.

Parents of children with disabilities face numerous difficulties in their daily life when caring for their child, along with feelings of worry and stress about their child’s long-term survival and well-being [2]. The difficulties that the parents face may include their ability to deal with day-to-day challenges, transformation of their social interactions with relatives and friends, and modification of their daily activities [2]. Parents of children with disabilities worry about their child’s acceptance in society, as well as the opportunities and resources that might not be available to them when caring for their child. As the child grows and their condition persists, parents become concerned about where and with whom their child will live when they are adults [3]. In addition, parents of children with disabilities experience emotions that are overwhelming and do not follow a predictable sequence. Their emotions can include sadness, anger, and frustration and be periodic in nature [4]. Multiple studies have aimed to measure chronic sorrow among parents of children with different health conditions. These include but are not limited to chronic illness and disabilities [4], autism [5,6], cancer [7], sickle cell disease [8], neurodevelopmental disorders [9], type 1 diabetes [10], and Down syndrome [11].

As a result, the concept of chronic sorrow emerged to explore the daily experiences of these parents. Roos [12] defined chronic sorrow as “a set of pervasive, profound, continuing, and recurring grief responses resulting from a significant loss or absence of crucial aspect of oneself (self – loss) or another living person (other – loss) to whom there is a deep attachment. The way in which the loss is perceived determines the existence of chronic sorrow. The essence of chronic sorrow is a painful discrepancy between what is perceived as reality and what continues to be dreamed of. The loss is ongoing since the source of the loss continues to be present. The loss is a living loss.”

### The Theoretical Framework of Chronic Sorrow

Chronic sorrow was initially proposed by psychiatrist Olshansky [13]. In his work with mentally challenged children, their parents, and family members, Olshansky observed that parents of children with disabilities display a widespread psychological response to the experience of having a child with disabilities [13]. The parents often had long-lasting, chronic sorrow because the loss they experienced lacked a clear end. It was also uncertain how long the loss would persist [14]. Parents who care for children with disabilities have described having profound emotional experiences. These emotions may be felt occasionally but they may not always be present. These intense emotions may include shock, disbelief, rage, irritation, a feeling of isolation, and a profound sense of sadness and loss. As a result, it is hypothesized that parents of children with disabilities are more likely to report experiencing persistent sadness [15].

Eakes et al [16] defined the variables of the theory of chronic sorrow as the following:

- Chronic sorrow: “ongoing disparity resulting from loss characterized by pervasiveness and permanence. Symptoms of grief recur periodically, and these symptoms are potentially progressive.”
- Loss: occurs because of the discrepancy between the “ideal” or “imagined” situation versus the lived experience and occurs in the early stages of the child’s diagnosis.
- Disparity: disparity follows the loss experience and can be classified as a trigger for chronic sorrow. The loss experience occurs in the early stages of the child’s diagnosis, whereas disparity reemerges as time passes, when the child does not meet his or her developmental milestones like his or her peers, such as attending school graduations and birthday parties.
- Management methods: strategies used by parents or individuals to minimize the impact of sorrow. These methods can be internal (personal and individualized methods) or external (support from health care workers or institutions).

Studies that have addressed chronic sorrow among parents of children with disabilities are very scarce, especially in Arabic. The Kendall Chronic Sorrow Instrument was found to be useful in measuring the chronic sorrow of mothers of a child with disabilities [17]. Fernandes et al [18] analyzed the theory of chronic sorrow and found that the theory is well-defined and its concepts captured the phenomena under study. When measuring its concepts, they found that the theory had high reliability.

It is worth noting that parents experiencing chronic sorrow are usually overlooked and remain unexamined by society. Parents of children with disabilities must navigate their emotions of sorrow and an unending sense of commitment while maintaining their level of functionality [19]. The objectives of this study are important because parents of children with disabilities report multiple negative outcomes such as psychological distress, a sense of isolation, a lack of financial support, and the absence of tools to help them manage their child’s condition [20].

Lastly, the theory of chronic sorrow can be used by nurses caring for parents of children with disabilities to help the parents identify and use management and coping strategies.

This descriptive study aims to explore chronic sorrow among parents of children with different types of disabilities in the Kingdom of Saudi Arabia. The objective of this study is to answer the following questions: With the use of chronic sorrow as a theoretical framework, what are the parental experiences of chronic sorrow in the context of caring for a child with a disability? Is there a relationship between the concepts of the theory of chronic sorrow, specifically between loss and disparity with chronic sorrow? Lastly, is there a relationship between the time elapsed since the child’s diagnosis and parental feelings of chronic sorrow?

## Methods

### Design

A quantitative, descriptive cross-sectional study design will be used to conduct this study exploring chronic sorrow among parents of children with disabilities. We will also identify the strength of the relationships between the triggers (loss experience and disparity) of chronic sorrow. Lastly, we will investigate the impact of time since the child’s diagnosis on parental feelings of chronic sorrow.

### Instrument

Data were collected for this study through a web-based questionnaire. The questionnaire and consent form were sent to the participants after obtaining institutional review board approval from King Abdullah International Medical Center.

The questionnaire was distributed electronically to participants via WhatsApp. The questionnaire consisted of parental demographic data (8 items), loss experience (5 items), disparity (3 items), chronic sorrow (9 items), and management of chronic sorrow (9 items). The chronic sorrow measurement was originally created by Kendall (Kendall Chronic Sorrow Instrument) [21], then the Arabic version of the same instrument was created by Baker et al [22], who confirmed that the internal consistency of the correlation coefficient of the scale was greater than 0.7, with a high degree of validity in the Loach method for all statements, with a score of 0.62. In addition, the Cronbach α was 0.8. All of the above parameters have met the psychometric properties of a valid and reliable instrument [22]. As a result, the Arabic version of the instrument was used for this study due to the proximity of the populations in both studies, both of which speak Arabic and have shared cultural characteristics and religious backgrounds.

For the measurement of chronic sorrow, every item in the scale had a total of 5 responses (5-point Likert scale), ranging from strongly disagree (score of 1 point), disagree (2 points), neutral (3 points), agree (4 points), to strongly agree (5 points). Scoring criteria were as follows: 1‐2 indicated a low score of chronic sorrow, 2‐3 indicated a moderately low score, 3‐4 indicated a moderately high score, and 4‐5 indicated a high score of chronic sorrow.

### Sample, Setting, and Data Collection

Recruitment of study participants was done using nonprobability convenience sampling. The tool targeted parents with a child with disabilities currently residing in Saudi Arabia. The participants who were eligible for this study fulfilled the following criteria: the mother, father, or primary caregiver of a child with a disability, the child had a confirmed medical diagnosis, the parent could speak and read Arabic, and parental age was above 18 years.

For this descriptive study, the significance level of α was set at 0.05 with a power of 0.8 to calculate the sample size and estimate power analysis. The sample size was estimated by G*Power software. This study will be the first to examine the use of chronic sorrow as a theoretical framework among Saudi parents who have a child with a disability. As a result, no previously reported effect size was available for use in the power analysis. Cohen [23] stated that in cases like our study where effect size has not been previously reported in the literature, the estimation of effect size should be based on logic and judgment. Therefore, a medium effect size of 0.30, a power of 0.80, and α significance level of 0.05 were used to guide our statistical analysis, with a sample size of 64.

Potential participants were recruited via invitation through WhatsApp, Facebook, and X (formerly known as Twitter). Upon filling out the questionnaire, the researcher identified parents who met the inclusion criteria and enrolled them in the study. If the participant agreed to fill out the questionnaire after reading the consent form, they were directed to the questionnaire page. Consent was obtained from participants electronically; if the participant agreed to fill in the questionnaire, he or she was directed to an electronic informed consent form. After reading the consent form, the participant was required to sign the form electronically by clicking the “I agree to participate” box, after which the questionnaire would appear.

We used Microsoft Forms for its easy accessibility and user-friendliness for the general population to promote data collection and reduce missing data.

### Data Analysis

Once data collection was complete, we entered the data into an Excel database for analysis. SPSS (version 30; IBM Corp) was used to analyze the data on a password-protected computer. Different types of analysis were proposed for this study. Descriptive statistics were calculated on all variables of interest, including means, standard deviations, frequencies, and percentages. Pearson correlation coefficients were used to examine the relationships among continuous variables. Statistical significance was based on the standard α level of .05.

Frequencies and percentages were used for analyzing descriptive data to evaluate the variables of chronic sorrow. Pearson correlations were used to analyze numerical data and assess the relationship between the triggers and the concept of chronic sorrow.

### Ethical Considerations

This study received ethics approval from the institutional review board of the King Abdullah International Medical Research Center (SP23J/144/09) on October 16, 2023, and the data collection lasted for 6 months.

Various measures were undertaken to maintain the privacy and confidentiality of participants. Although there was minimal risk involved, all measures to protect participants’ information were taken. Participants’ responses to each survey item were not shared with other participants or individuals not associated with the research project. By accessing and completing the survey, participants gave their consent to take part in the study. Participation in the study was voluntary. Participants could withdraw from the study at any time. No compensation was provided to the participants.

## Results

### Participant Characteristics

As Table 1 shows, a total of 89 participants completed the survey. Of these participants, 78% (n=69) were mothers of a child with disabilities. Most of the participants (n=42, 48%) were aged 40-49 years. Many of the participants were caring for a child with a permanent disability (n=79, 89%) and most participants had a university level of education (n=54, 61%). Most of the children had been diagnosed for less than 1 year (n=45, 51%) and Down syndrome was the most reported disability (n=39, 44%).

**Table 1.** The sample demographic characteristics (N=89).

Demographic variable	Participants, n (%)
**People completing the survey**	
Mother	69 (78)
Father	5 (6)
Nonparent	15 (17)
**Age group (years)^a^**	
20‐29	5 (6)
30‐39	20 (23)
40‐49	42 (48)
50‐59	16 (18)
60‐65	4 (5)
**Permanence of the child’s disability**	
Temporary disability	10 (11)
Permanent disability	79 (89)
**Educational level**	
Primary school	4 (4)
Intermediate school	5 (6)
High school	26 (29)
University	54 (61)
**Time since the child was diagnosed with a disability (years)**	
Less than 1	45 (51)
2‐3	11 (12)
4‐9	25 (28)
More than 10	8 (9)
**Nature of the child’s disability**	
Down syndrome	39 (44)
Autism and attention-deficit/hyperactivity disorder	15 (17)
Mental retardation	8 (9)
Physical disability	21 (24)
Undisclosed disability	6 (7)

a There were 87 responses for this question (ie, 2 participants did not respond).

### Descriptive Statistics of the Theory of Chronic Sorrow

The data in the following 5 sections illustrate the descriptive statistics of the participants toward the concepts of the theory of chronic sorrow, which is used to measure the following aspects: the triggers of loss experience and disparity, chronic sorrow, internal management, and external management.

### Descriptive Statistics for Loss Experience

As shown in Table 2, the reported score of the loss experience that parents of children with disabilities face was a mean of 3.3 (SD 1.10). Of all the variables measuring loss experience, feeling sad when thinking about the child’s disability was the highest (mean 3.9, SD 1.24) and a sense of overwhelming sorrow scored the lowest (mean 2.8, SD 1.40). The results showed that parents with children with disabilities reported moderately high loss experience.

**Table 2.** Descriptive statistics of loss experience (N=89).

Loss experience	Score, mean (SD)
It feels like the disability happened to me	3.5 (1.18)
Sorrow feelings when I think about my child’s disability	3.9 (1.24)
The sorrow feeling remains the same as the day of the diagnosis	3.3 (1.43)
Urge to cry when I remember the disability	3.3 (1.42)
Overwhelming sorrow	2.8 (1.40)
Total score of loss experience	3.3 (1.10)

### Descriptive Statistics for Disparity

As shown in Table 3, the total score of the concept of disparity reported by parents of children with disabilities had a mean of 3.3 (SD 1.06). The highest score among the disparity variables was the feeling of sorrow and sadness parents face when their child cannot meet developmental milestones, often associated with significant events or dates including birthdays, with a mean score of 3.8 (SD 1.30). On the other hand, the lowest score reported was the feeling of sorrow when thinking about their child without the disability (mean 2.7, SD 1.28). According to this study, the feeling of disparity experienced by parents of children with disabilities is moderately high, which is aligned with the experiences of loss.

**Table 3.** Descriptive statistics of disparity (N=89).

Disparity	Score, mean (SD)
Sorrow emerges when I remember my child’s disability	3.3 (1.29)
I feel sorrow about things that do not matter for parents with healthy children (such as birthdays and entering school)	3.8 (1.30)
I feel sorrow when I picture my life without my child’s disability	2.7 (1.28)
Total score of disparity	3.3 (1.06)

### Descriptive Statistics for Chronic Sorrow

As shown in Table 4, the total score of the chronic sorrow reported by parents of children with disabilities had a mean of 3 (SD 0.87). Of the variables measuring chronic sorrow, the variable of sorrow and its nature of coming and going (ie, its periodic nature) had the highest score (mean 3.6, SD 1.16), along with reported energy to handle sorrow (mean 3.6, SD 1.11). On the other hand, the least reported variable within the chronic sorrow concept was parents reporting that their life is not what they imagined due to their child’s disability (mean 2.6, SD 1.33), followed by feeling that parental desires and goals do not match what life has given them (mean 2.7, SD 1.32). Overall, parents reported moderately high levels of chronic sorrow, indicating that these feelings of sorrow are manageable and can be reduced or that its cyclic nature can be spaced out.

**Table 4.** Descriptive statistics of chronic sorrow (N=89).

Chronic sorrow	Score, mean (SD)
Sorrow regarding my child’s disability comes and goes	3.6 (1.16)
I feel like my child’s disability makes me give up some aspects of my life	3.4 (1.40)
I feel like I do not have control over my life	2.9 (1.36)
I feel like my life is not like I imagined due to my child’s disability	2.6 (1.33)
The feelings of sorrow can transform into feelings of loneliness	2.8 (1.38)
I feel like I have energy to handle life’s stressors	3.6 (1.11)
Sudden changes in my child’s disability have led to exhaustion	3.0 (1.26)
I think that what I desired does not align with what life has given me	2.7 (1.32)
I feel older due to my child’s disability	2.9 (1.34)
Total score of chronic sorrow	3.0 (0.87)

### Descriptive Statistics of Internal Management of Chronic Sorrow

Table 5 presents the descriptive statistics of how parents of children with disabilities view the effectiveness of internally managing their child’s disability as a means to overcome chronic sorrow. The total score of internal management had a mean of 4.6 (SD 0.33), which is a high score, highlighting the important role of internal management of chronic sorrow. Of all the variables measuring the internal management of chronic sorrow, accepting the child’s disability as destined by God (also known in the Islamic religion as fatalism) demonstrated the highest score with a mean of 4.8 (SD 0.50), followed by the availability of resources that parents need to manage their child’s disability (mean 4.7, SD 0.47). These were followed by feeling better when parents are more aware and can make independent decisions about the child’s disability (mean 4.4, SD 0.62). The results of this study showed that internal management of a child’s disability is crucial for reducing chronic sorrow and empowering the parents of children with disabilities to minimize their episodes of chronic sorrow, allowing them to provide the best care for their child.

**Table 5.** Descriptive statistics of internal management of chronic sorrow by parents of children with disabilities.

Internal management of chronic sorrow	Score, mean (SD)
I feel better when I am more aware of my child’s disability	4.4 (0.62)
Making independent decisions about my child’s disability is empowering for me	4.4 (0.62)
Accepting my child’s disability as destined by God makes me feel better	4.8 (0.50)
The availability of resources (eg, financial and therapeutic) for my child induces a sense of relief	4.7 (0.47)
Total score of internal management	4.6 (0.33)

### Descriptive Statistics of External Management of Chronic Sorrow

Table 6 shows results related to parents’ external management of chronic sorrow, with the overall utilization of external management displaying great importance (mean 4.5, SD 0.42). Of all the variables measuring external management of chronic sorrow, feeling better when social support is provided displayed the highest score (mean 4.7, SD 0.55), followed by feeling better when the community is kinder and more considerate (mean 4.5, SD 0.56). The results of this study showed that both external and internal management of chronic sorrow are important tools that parents need to use and navigate to manage the periodic and unpredictable nature of chronic sorrow.

**Table 6.** External management of chronic sorrow by parents of children with disabilities.

External management of chronic sorrow	Score, mean (SD)
I feel better when doctors and nurses provide more information about my child’s condition	4.4 (0.64)
I feel better when support is provided by family and the community	4.7 (0.55)
I feel better when I am allowed to express my emotions freely and without judgment	4.3 (0.71)
I feel better when I believe that we as a family have created a coping strategy that is specific to us	4.4 (0.62)
I feel better when more people in the community are considerate and kind to me and my child	4.5 (0.56)
Total score of external management of chronic sorrow	4.5 (0.42)

### Association Between Chronic Sorrow and Parental Loss Experience and Disparity

Table 7 shows the Pearson correlation between chronic sorrow and the sense of loss experience and disparity. The study results showed a strong correlation between chronic sorrow and the concepts of disparity and loss.

As shown in Table 7, chronic sorrow is strongly and positively correlated with loss experience (*r*=0.765; *P*<.001). In addition, the same strong correlation emerged between chronic sorrow and disparity (*r*=0.765; *P*<.001). Lastly, another positive and strong correlation emerged between the concept of loss experience and disparity (*r*=0.791; *P*<.001).

**Table 7.** Pearson correlation between chronic sorrow and loss experience and disparity.

Variable	Chronic sorrow	Loss experience	Disparity
**Chronic sorrow**			
*r*	1	0.765	0.765
*P* value	—^a^	<.001	<.001
**Loss experience**			
*r*	0.765	1	0.791
*P* value	<.001	—	<.001
**Disparity**			
*r*	.765	0.791	1
*P* value	<.001	<.001	—

a Not applicable.

### Association Between Chronic Sorrow and Time Since Diagnosis of the Child’s Disability

Table 8 illustrates the results of the Pearson correlation between chronic sorrow and the amount of time elapsed since parents received their child’s diagnosis. The correlation was negative and very weak (*r*=–0.009; *P*<.93), indicating that, as time passes, the feelings of chronic sorrow among parents do not subside. This study showed an inverse yet very weak relationship between time since diagnosis and the chronic sorrow score, indicating that time passing does not play a role or have an impact on the recurrent nature of chronic sorrow. It is also worth mentioning that an increase in chronic sorrow as time passes was not indicated. Therefore, health care workers need to assess parents for chronic sorrow whether the diagnosis was received recently or a long time ago, highlighting the importance of multiple interventions to manage and coexist with chronic sorrow.

**Table 8.** Pearson correlation between the concept of chronic sorrow and time since diagnosis.

Variable	Chronic sorrow	Time since child’s diagnosis
**Chronic sorrow**		
*r*	1	–0.009
*P* value	—^a^	.94
**Time since child’s diagnosis**		
*r*	–0.009	1
*P* value	.94	—

a Not applicable.

## Discussion

### Principal Findings

Literature examining chronic sorrow among parents with a child with a disability was scarce in the international and Saudi literature alike. Therefore, this study was designed to examine the application of the theory of chronic sorrow by Olshansky [13] to Saudi parents with a child with a disability. The study examined the major variables in the theory including loss experience, disparity, chronic sorrow, and internal and external management. In addition, we attempted to examine the relationships between these variables and whether time since diagnosis decreased the sense of chronic sorrow. The results of the study showed that parents reported a moderately high level of loss experience, disparity, and chronic sorrow. On the other hand, management methods (internal or external) played a crucial role for these parents in adapting to and managing their child’s disability. In addition, the reported concepts of loss experience and disparity had a moderately strong relationship with the feelings of chronic sorrow that the parents were experiencing. Lastly, time elapsed since the child’s diagnosis did not have an inverse relationship with the feeling of chronic sorrow, indicting that time passing does not play a role in the feeling of chronic sorrow.

Parents of children with disabilities have experienced loss, which is different for every parent; in addition, different parents have different abilities to navigate emotions. Loss experience can be interpreted as the loss of the ideal child [24]. This study found a moderately high level of loss experience and that its consequences can trigger the feeling of chronic sorrow. Loss experience among parents of children with disabilities has been reported in previous studies, reflecting the results of our study despite cultural and religious differences between the study populations. A study by Phillips [25] examined loss among parents of children with disabilities, viewing loss as a consequence that they are not familiar with, and its ability to impact the family’s well-being. Studies by Fernandez et al [18] and Fernández-Alcántara et al [24] examined loss and grief (eg, related to loss of the ideal child, a traumatic experience, and shock) in the same population, and the concept of the theory of chronic sorrow emerged there as well. Loss experience among parents is a crucial step that parents undergo to explore their chronic sorrow, eventually leading to acceptance and hope for their child’s future.

Disparity is one of the major concepts of the theory of chronic sorrow. It follows the feelings of loss experience and is triggered by certain developmental milestones that parents of children with disabilities observe in healthy children [8]. Multiple studies have illustrated the presence of disparity within parents; those results were congruent with the results of this study. A study by Nikfarid et al [26] examined chronic sorrow in parents of children who were diagnosed with cancer, while a study by Olwit et al [8] investigated chronic sorrow in parents with children diagnosed with sickle cell disease. In both of these studies, parents mentioned the presence of disparity and its role in triggering chronic sorrow, contributing to its ongoing nature. Lastly, a study by Masterson [27] explored parental chronic sorrow and disparity when a child with cerebral palsy becomes an adult. The study showed that mothers still experienced disparity, which triggered a loss of hope as the child aged without meeting milestones.

Chronic sorrow among parents with a child with a disability is different from the normal grieving process or sadness because it is characterized by its cyclic nature, and it can be triggered by feeling of loss and disparity [13]. Results from studies examining chronic sorrow among parents with children with disabilities have been similar to and consistent with the results of this study. The theory of chronic sorrow explains the realistic feelings and emotions encountered by these parents and that these feelings do not manifest the same way as grief or a single loss because they can appear after parental acceptance of the disability [26-29]. Studies by Olwit et al [8] and Hobdell [30] found that chronic sorrow among parents needs to be assessed by health care workers as it has the potential to affect parental health and parental management of their child’s disability. In parents, chronic sorrow may present as reduced social interaction, psychological distress, anger, and even guilt [20,31,32].

Internal management of chronic sorrow that is effective and used by parents plays a crucial role in minimizing the recurrence of chronic sorrow episodes, particularly if it is used correctly [18]. This study illustrates that fatalism (accepting God’s destiny regarding the child’s disability) represents the most effective internal management strategy for chronic sorrow. Multiple studies have displayed similar results in spite of different religious or spiritual beliefs among the study participants. Studies by Mikołajczuk and Zielińska-Król [33] and Pillay et al [34] reported that parents of children with disabilities found that religion and accepting God’s destiny were effective coping mechanisms that improved parental resiliency, acceptance, and management of daily challenges. The participants of this study were predominantly from the Islamic religion, and the concept of fatalism as a means to internally manage chronic sorrow was most reported in this population. Fatalism in the Islamic religion is the belief that the future of man is already determined by God and cannot be swayed or changed whether the destiny is good or bad, and man should accept god’s destiny and surrender to his will. Khan et al [35] and Othman et al [36] studied Muslim parents of children with disabilities and their results—in which parents found comfort, hope, and resiliency when integrating religion and spirituality when caring for their children—are congruent with this study.

Its important to note that this study is the first to use the theory of chronic sorrow among Saudi parents of children with disabilities, and it is crucial to incorporate religion and the concept of fatalism into this framework to fully capture the phenomena of chronic sorrow as the role of religion in navigating emotions is important in this population. The role of religion and belief in this context was previously reported in the literature by Alqunaibet [37] and Çaksen [38], who illustrated that religion and spirituality play an important role in helping parents and caregivers manage sorrow and stress.

Our questions regarding the external management of chronic sorrow aimed to capture the most efficient method used by parents. The results of the study identified social support (from family and the community in particular) to be key to externally managing chronic sorrow. The results of the study correspond with those of Ha et al [39] and Felizardo et al [40], who investigated the role of social support from family and the community, which yielded similar results to this study. Mantri‐Langeveldt et al [41] conducted a scoping review to illustrate the important role of family support for parents with children with disabilities, reaffirming the importance of social support measurement by health care professionals.

This study identified a strong positive relationship between chronic sorrow and loss. The loss expressed by parents of children with disabilities is known as ambiguous loss as it is not definitive and not clearly visible as the child is alive, though not experiencing normal development. In addition, the loss associated with chronic sorrow in this population does not provide closure; the loss of the ideal child cannot be mourned because that child never existed [42]. Brown [43] conducted a study to capture the importance of sorrow and grieving in the healing process for mothers of children with intellectual disabilities. The results are similar to this study, as mothers experienced loss specifically after diagnosis and felt loss for their child and for their role as mothers. Senenac et al [44] described the parental experience of having a child with disabilities and the results identified that the themes of sorrow and loss are associated, along with other emerging concepts such as stigma and guilt.

This study identified a strong and positive relationship between chronic sorrow and disparity. Disparity, unlike loss, reemerges as time passes and the parents tangibly observe the inability of their child to meet developmental milestones like their peers, which triggers feelings of chronic sorrow [17]. Claassens et al [4] examined parental chronic sorrow among parents with children with disabilities. The results of the review yielded multiple findings, one of which was the strong relationship between chronic sorrow and disparity. The review identified events that parents undergo that trigger the resurgence of chronic sorrow and their role in reminding parents about the disparity and gap between the “idealized and hoped-for child” and “the ill or disabled child” [4]. Multiple studies have found that the same phenomenon is seen with disparity and its role in triggering chronic sorrow in parents caring for children with different types of disabilities or diseases that require caregiving, such as autism spectrum disorder [45], sickle cell disease [8], and cerebral palsy [46].

This study examined the relationship between time elapsed since the child received their diagnosis and chronic sorrow among parents. The results of this study have shown that there is no relationship between time passing and chronic sorrow, indicating that time does not play a role in reducing or increasing feelings of chronic sorrow. The results of this study correspond with the literature. A study by Fernandez et al [18] found that loss and sorrow persist over time and therefore emotional interventions are crucial for the parents of children with disabilities. Fernández‐Ávalos et al [47] rationalized this phenomenon by explaining that parents are faced with daily challenges and worries when caring for their child. As a result, these challenges influence their perception of managing chronic sorrow and their quality of life. Therefore, delivering early interventions and realistic expectations of the child’s development and disease prognosis are crucial, along with providing emotional care for parents. These interventions will play a role in managing chronic sorrow over time and eventually lead to acceptance, resilience, and enhanced mental health [48-50].

### Limitations

This study collected data from parents primarily residing in Jeddah, Saudi Arabia. Therefore, the results of the study might not be generalizable to other countries or cultures. Another limitation was the type of disability that the children were diagnosed with, as much of the data were collected from parents with children with Down syndrome (44%), which limits generalizability to other types of disabilities that can be more severe or require less caregiving.

This study used nonprobability convenience sampling to recruit the parents of children with disabilities. This type of sampling has the potential to limit generalizability and therefore lacks external validity. It is worth noting that the study was conducted in Jeddah City, Saudi Arabia, and the majority of the participants reside in the western region of the country; we did not examine chronic sorrow in more rural areas of Saudi Arabia or other Middle Eastern countries that share multiple cultural aspects, which limits the generalizability of the study findings.

Another limitation was the study’s cross-sectional design. Cross-sectional study designs create ambiguity about the direction of the causal relationship between study variables. Lastly, to overcome time and financial constraints that the researchers were faced with, data were self-reported via a web-based questionnaire. Therefore, the conditions in which parents filled out the survey were not controlled nor objectively measured.

### Conclusion and Recommendation

The aim of this study was to examine the experience of parents of children with disabilities with chronic sorrow in Saudi Arabia using the theory of chronic sorrow. We found moderately high levels of loss experience, disparity, and feelings of chronic sorrow among study participants. Therefore, the results of this study highlight the importance of chronic sorrow management and the creation of interventions to enhance parental mental health and well-being. In addition, internal management of chronic sorrow played a crucial role. In particular, we demonstrated the importance of the concept of fatalism and religious and spiritual beliefs among Arab Muslim parents of children with disabilities. With regard to external management, parents reported that family and community support were pivotal for the management of chronic sorrow, highlighting the social responsibility of all members of the community to provide support to both parents and children. This study also identified a strong and positive relationship between the concepts of the theory of chronic sorrow, specifically between the identified triggers of loss and disparity and chronic sorrow. Lastly, the study results found no relationship between feelings of chronic sorrow and time elapsed since the parents received their child’s diagnosis. Given the results of this study, parents of children with disabilities need to be periodically screened for chronic sorrow as these feelings and coping strategies need to be addressed. Parental acceptance of their child’s disability does not signify the absence of chronic sorrow, as it is part of the normal grieving process, but rather the anticipation of the triggers of chronic sorrow. The use of management methods, both internal and external, is essential for promoting parental mental health and child health outcomes.

This study has the potential to provide the foundation for future studies navigating and investigating chronic sorrow among parents with a children with a disability. Future studies could include an investigation of chronic sorrow based on the severity of the disability among parents caring for adult children with a disability and could be expanded to extended family members and caregivers.

## Abbreviations

CS: chronic sorrow
